# Astrocyte elevated gene-1 is associated with metastasis in head and neck squamous cell carcinoma through p65 phosphorylation and upregulation of MMP1

**DOI:** 10.1186/1476-4598-12-109

**Published:** 2013-09-24

**Authors:** Yi-Ping Wang, I-Ju Liu, Chiung-Pin Chiang, Han-Chung Wu

**Affiliations:** 1 Graduate Institute of Clinical Dentistry, School of Dentistry, National Taiwan University, Taipei, Taiwan; 2 Institute of Cellular and Organismic Biology, Academia Sinica, Taipei, Taiwan; 3 Department of Dentistry, National Taiwan University Hospital, Taipei, Taiwan; 4 Graduate Institute of Oral Biology, School of Dentistry, National Taiwan University, Taipei, Taiwan

**Keywords:** Astrocyte elevated gene-1 (AEG-1), Head and neck squamous cell carcinoma (HNSCC), Metastasis, Matrix metalloproteinase 1 (MMP1), p65

## Abstract

**Background:**

The survival rate of head and neck squamous cell carcinoma (HNSCC) at advanced stage is poor, despite contemporary advances in treatment modalities. Recent studies have indicated that astrocyte elevated gene-1 (AEG-1), a single transmembrane protein without any known functional domains, is overexpressed in various malignancies and is implicated in both distant metastasis and poor survival.

**Results:**

High expression of AEG-1 in HNSCC was positively correlated with regional lymph node metastasis and a poor 5-year survival rate. Knockdown of AEG-1 in HNSCC cell lines reduced their capacity for colony formation, migration and invasion. Furthermore, decreased tumor volume and metastatic foci were observed after knockdown of AEG-1 in subcutaneous xenografts and pulmonary metastasis assays *in vivo*, respectively. We also demonstrated that AEG-1 increased phosphorylation of the p65 subunit of NF-κB, and regulated the expression of MMP1 in HNSCC cells. Moreover, compromised phosphorylation of the p65 (RelA) subunit of NF-κB at serine 536 was observed upon silencing of AEG-1 in both HNSCC cell lines and clinical specimens.

**Conclusion:**

High expression of AEG-1 is associated with lymph node metastasis and its potentially associated mechanism is investigated.

## Background

Head and neck squamous cell carcinoma (HNSCC) poses a grave threat to public health in Melanesia, South-Central Asia, and Central and Eastern Europe, with 263,900 new cases and 128,000 HNSCC related deaths reported worldwide annually [1]. This cancer usually arises within the mucosa lining the upper aerodigestive tract, with oral cavity, oropharynx, hypopharynx and larynx being the four most common affected sites. Regional lymph node metastasis, which is a common feature, is present in approximately two thirds of patients with advanced stage HNSCC. Increased number of lymph nodes with metastatic lesions and the presence of extranodal spread are strong predictors for distant metastasis and poor survival of the patient [2]. Despite recent advances in oromaxillofacial surgery and combination treatment using either EGFR-targeting antibodies or tyrosine kinase inhibitors, there has been little improvement in the survival of patients with metastatic HNSCC [3-5]. As such, there is an urgent need to identify new predictive parameters for lymph node metastasis and novel therapeutic targets for HNSCC.

Astrocyte elevated gene-1 (AEG-1), also known as metadherin (MTDH) or LYsine-RIch CEACAM1 co-isolated (LYRIC), is a 582 amino acid residues type II transmembrane protein without any known functional domains. It has emerged as a novel oncoprotein essential for malignant progression in various types of human cancers [6-13]. The amino acid sequence of AEG-1 possesses three nuclear localization signals, and ubiquitination of AEG-1 determines the subcellular region to which it is transported [14] through an as yet undelineated mechanism. Brown et al. used phage display to identify AEG-1 as a receptor that mediates adhesion of murine mammary tumor cells to lung endothelial cells and promotes lung metastasis [15]. Membranous AEG-1 has been shown to enhance adhesion of tumor cells to pulmonary microvascular endothelial cells [9]. The major signaling cascades activated by AEG-1 are the PI3K and NF-κB pathways, [6,16,17] and AEG-1 has recently been proposed to physically interact with AP1, SND-1, and the p65 subunit of NF-κB [18-20]. However, the direct effects on the associated proteins after the binding of AEG-1 remain unclear. Expression of AEG-1 is increased by TNF-α and HIV infection in astrocytes, whereas microRNA-375 (miR375) is a negative regulator of AEG-1 [13,21,22]. Mounting evidence suggests that AEG-1 confers pleiotrophic aggressive phenotypes in malignant neoplasms, especially with respect to invasion and metastasis. Nonetheless, the definitive link between the expression of AEG-1 and its prognostic value in HNSCC patients still needs to be established with a large cohort of clinical specimen, while its underlying molecular mechanisms need to be elucidated. Since the presence of metastatic lesions has a negative impact on the prognosis and morbidity of HNSCC patients, it prompts us to investigate the biological role of AEG-1 in this disease entity.

In the current article, we report that AEG-1 is overexpressed in a majority of clinical specimens of oral squamous cell carcinoma (OSCC, a subset of HNSCC), and its expression is positively associated with both the presence and the degree of lymph node metastasis. Knockdown of AEG-1 also decreases the aggressiveness of HNSCC cell lines both *in vitro* and *in vivo*. As far as we know, this is the first study to demonstrate that AEG-1 modulates the phosphorylation at serine 536 of the p65 subunit of NF-κB in HNSCC, which in turn regulates the production of MMP1 by manipulating the binding of NF-κB to its promoter region.

## Results

### High AEG-1 expression in OSCC is associated with regional lymph node metastasis and unfavorable 5-year survival

**Figure 1:**
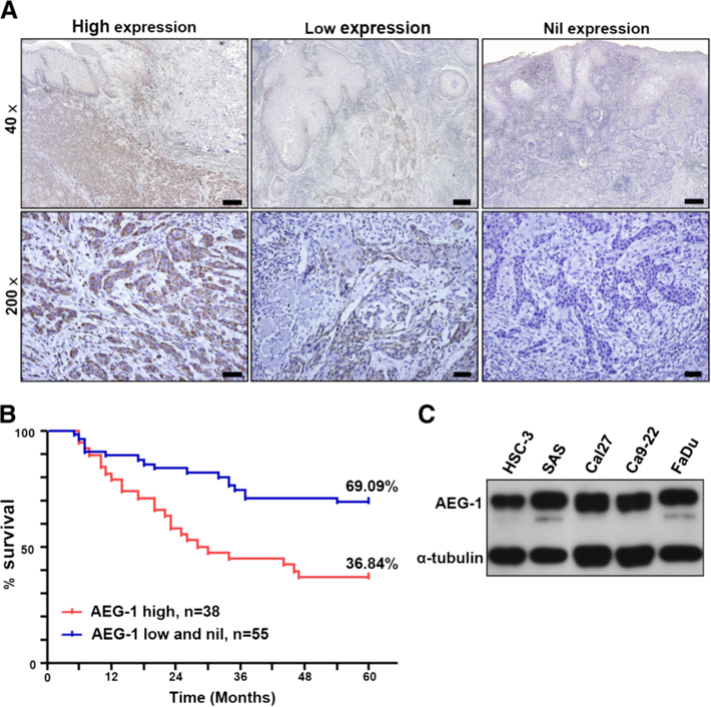
**AEG-1 expression in clinical specimens of OSCC and cell lines of HNSCC. (A)** immunohistochemical staining of formalin-fixed, paraffin embedded OSCC specimens. Scale bar: 40 ×, 300 μm; 200 ×, 45 μm. **(B)** Kaplan-Meier 5-year survival analysis of 93 cases of OSCC segregated by expression status of AEG-1 protein. **(C)** AEG-1 protein expression in HNSCC cell lines.

Immunohistochemical analysis of AEG-1 revealed high expression of AEG-1 in 40.86% (38 out of 93) of examined OSCC clinical specimens. AEG-1 was primarily located in the cytoplasm (the perinuclear region, in particular) of the neoplastic cells, and focal nuclear stains were also observed. In tumors with low AEG-1 expression, the majority of AEG-1-positive cells were found at the peripheral cells of the tumor nests, and not in the more-differentiated malignant cells (Figure 1A). In addition, no positive signal of AEG-1 was discerned in all 30 cases of uninflamed normal oral mucosa. Of the clinical parameters examined, late clinical stage (*p* = 0.01) and positive regional nodal metastasis (*p* < 0.001) were found to be significantly correlated to AEG-1 expression (Table 1). Futhermore, advanced lymph node metastasis (N2 and N3) is more common in the high AEG-1-expressing group (*p* = 0.012, Additional file 1: Table S1). The incidence of distant metastasis was also elevated (albeit not significantly) in the high AEG-1-expressing group, as compared to that in the low and nil AEG-1-expressing groups (10.53% and 1.83%, respectively). Furthermore, a statistically significant reduction in the 5-year disease-specific survival rate was observed in the high AEG-1-expressing group as compared to that in the low and nil AEG-1-expressing groups (36.84% versus 69.09%, log-rank test *p* = 0.0014, Figure 1B). These results imply that AEG-1 is associated with metastasis of OSCC and may serve as a negative prognostic factor for survival.

**Table 1:** Clinicopathological correlation with AEG-1 in 93 cases of OSCC

**Parameter**	**AEG-1 expression status**		**Fisher’s exact test**
	**Low**	**High**	
	**No. (%)**	**No. (%)**	***p *value**
**Gender**			
Male	46 (83.64%)	33 (86.84%)	0.773
Female	9 (16.36%)	5 (13.16%)	
**Age**			
>50 y/o	36 (65.45%)	20 (52.63%)	0.282
<50 y/o	19 (34.55%)	18 (47.37%)	
**Location**			
Buccal mucosa	22 (40.00%)	18 (47.37%)	0.307
Gingiva	11 (20.00%)	3 (7.89%)	
Floor of the mouth	1 (1.81%)	2 (5.26%)	
Lip	0 (0%)	2 (5.26%)	
Tongue	19 (34.55%)	12 (31.58%)	
Palate	2 (3.64%)	1 (2.64%)	
**Stage**			
I + II	30 (54.55%)	10 (26.32%)	0.01
III + IV	25 (45.45%)	28 (73.68%)	
**T**			
T1 + T2	31 (56.36%)	22 (57.89%)	1.000
T3 + T4	24 (43.64%)	16 (42.11%)	
**N**			
N0	47 (85.45%)	17 (44.74%)	<0.001
N1 + N2 + N3	8 (14.55%)	21 (55.26%)	
**M**			
M0	54 (98.18%)	34 (89.47%)	0.155
M1	1 (1.82%)	4 (10.53%)	
**Recurrence**			
Negative	45 (81.82%)	30 (78.95%)	0.793
Positive	10 (18.18%)	8 (21.05%)	
**Differentiation**			
Well	48 (87.27%)	27 (71.05%)	0.064
Moderate/poor	7 (12.73%)	11 (28.95%)	
**Alcohol**			
Negative	24 (43.64%)	10 (26.32%)	0.125
Positive	31 (56.36%)	28 (73.68%)	
**Betel nut**			
Negative	16 (29.09%)	6 (15.79%)	0.214
Positive	39 (70.91%)	32 (84.21%)	
**Cigarette**			
Negative	14 (25.45%)	8 (21.05%)	0.805
Positive	41 (74.55%)	30 (78.95%)	

### AEG-1 knockdown reduced the aggressiveness of HNSCC cell lines *in vitro*

**Figure 2:**
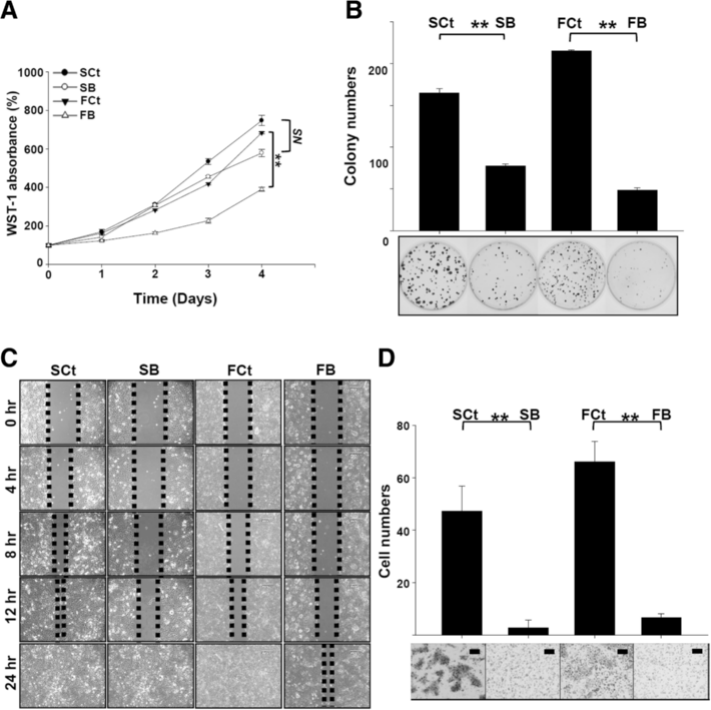
**Impact of AEG-1 knockdown on the function of HNSCC cell lines. (A)** WST-1 cell proliferation assay. **(B)** colony formation assay. **(C)** wound-healing migration assay. Initial gap: 500 μm. **(D)** transwell Matrigel invasion assay. All values are the average of three independent experiments. SB, AEG-1 knock-down SAS cells. FB, AEG-1 knock-down FaDu cells. SCt, SAS cells transfected with scrambled control shRNA. FCt, FaDu cells transfected with scrambled control shRNA. All data were expressed as mean ± SEM; n = 3. NS, not significant (*p* > 0.05); **, *p* < 0.01. Scale Bar: 130 μm.

To establish an *in vitro* platform for elucidation of the biological function of AEG-1 in HNSCC cell lines, we examined the expression status of AEG-1 in several cell lines generated from HNSCC. Western blots revealed that AEG-1 was ubiquitously expressed in all HNSCC cell lines tested (Figure 1C). We subsequently generated stable clones of SAS and FaDu cells expressing AEG-1-shRNA-B, in which AEG-1 mRNA and protein are efficiently suppressed (SB cells and FB cells, respectively; Additional file 2: Figure S1). Although marginal inhibition of cellular proliferation was observed after knockdown of AEG-1 in SB cells, FB cells demonstrated remarkable reduction in proliferation (77.39%, *p* = 0.056 and 57.07%, *p* < 0.001 on average as compared to the corresponding control, respectively, by day 4 after seeding, Figure 2A). A dramatic reduction of colonies was also observed in both SB and FB cells, as compared to that in the relevant control (77 versus 165 colonies and 48 versus 215 colonies on average for the SAS groups and FaDu groups, respectively, Figure 2B). Delayed wound healing and reduced Matrigel penetration were observed in AEG-1 knockdown cells (Figures 2C and D, respectively). At 12 hours after removal of the inserts, cells covered 96.9% of the visualized field area in the SCt group, but only 74.5% in the SB group (66% and 64.4% at the initial time point, respectively, Figure 2C). For the FaDu group at 12 hours, 85.9% and 78.5% of the areas were occupied by FCt and FB cells, respectively (initial, 70.8% and 70.1%, Figure 2C). The number of penetrated cells in AEG-1 knockdown cells was about 10% of the relevant control, for both SAS and FaDu cells (Figure 2D). These observations suggest that AEG-1 contributes to aggressive phenotypes of HNSCC cells, particularly with regards to their migration and invasion capacities.

### AEG-1 knockdown reduces tumor volume and pulmonary metastatic nodules of HNSCC cell lines *in vivo*

**Figure 3:**
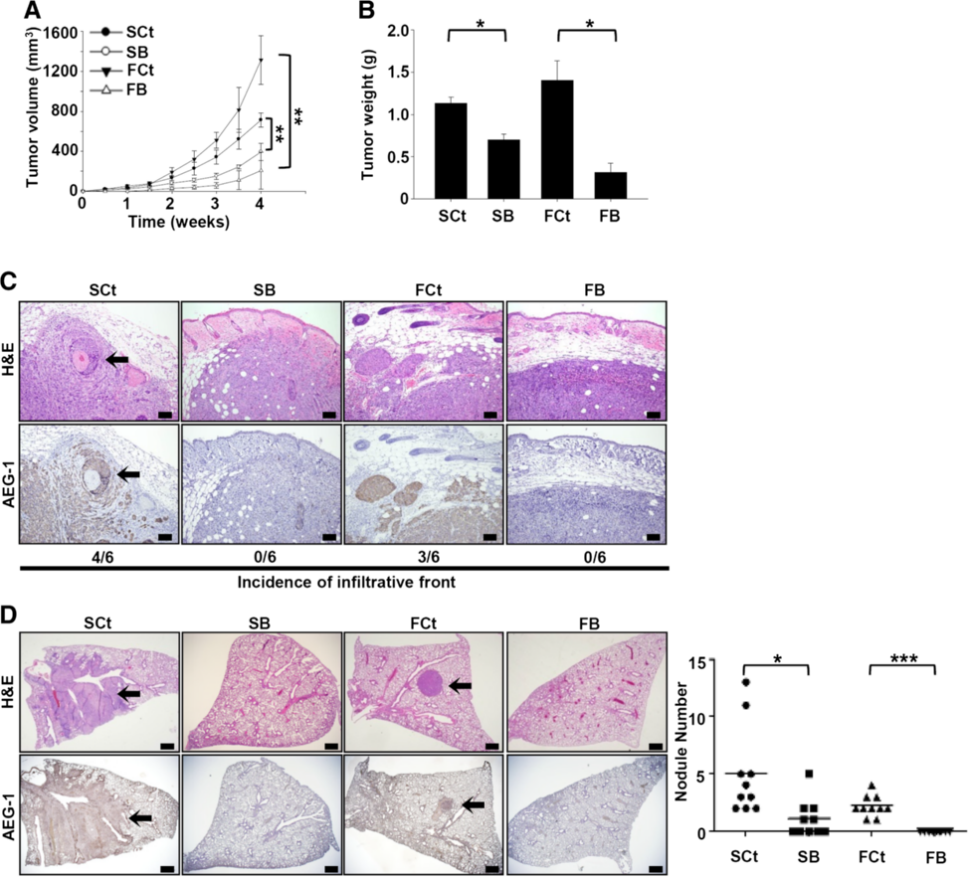
**AEG-1 knockdown compromises tumorigenecity (n = 6) and pulmonary metastasis (n = 10) of SAS and FaDu cells. (A)** time-course plot of tumor volumes. **(B)** tumor weights at the end-point. All data were expressed as mean ± SEM. *, *p* < 0.05; **, *p* < 0.01. **(C)** microscopy images of xenograft tumors at the end-point. Note the infiltrated invasion fronts in the SCt and FCt groups and the expansile tumor borders in the SB and FB groups. Arrow, focus of perineural invasion. Upper row, H&E stain; lower row, immunohistochemical stain with Lyric 4–7. Scale bar, 100 μm. **(D)***In vivo* lung metastasis assay. Arrow, metastatic focus. Upper row, H&E stain; lower row, immunohistochemical stain with anti- AEG-1 antibody. Scale bar, 500 μm.

To evaluate the biological impact of AEG-1 knockdown on HNSCC cell lines *in vivo*, subcutaneous xenografts were implanted into the flanks of Nod/SCID mice. Consistent with the results acquired *in vitro*, the volume of tumors arising from AEG-1-knockdown cells was smaller than those arising from the relevant control cells at all time points examined, with the suppression effect being more evident in FaDu cell lines (394.99 versus 714.71 mm^3^ in the SAS group and 207.70 versus 1314.33 mm^3^ in the FaDu group at the end point, Figure 3A). The tumor weight at the end-point of the experiment was also decreased in the AEG-1-knockdown groups as compared to that in the control groups (Figure 3B). Histopathological examination of harvested xenografts revealed infiltrating invasion fronts in a pattern of discrete cell nests in four out of six tumors from SCt cells and in three out of six tumors from FCt cells (Figure 3C). However, all xenografts from SB and FB cells assumed an expansile pattern of growth. Furthermore, perineural encroachment by the tumor cells was evident in two xenografts from the SCt cells. The numbers of pulmonary metastatic foci in the AEG-1-knockdown groups were also remarkably less than those of the corresponding control groups, and the size of the metastatic foci from SB cells was smaller than those observed in the SCt group (Figure 3D). These *in vivo* observations are consistent with the findings in clinical specimens and further support the hypothesis that AEG-1 is involved in the metastatic cascade of HNSCC.

### AEG-1 suppression downregulates MMP1 production

**Figure 4:**
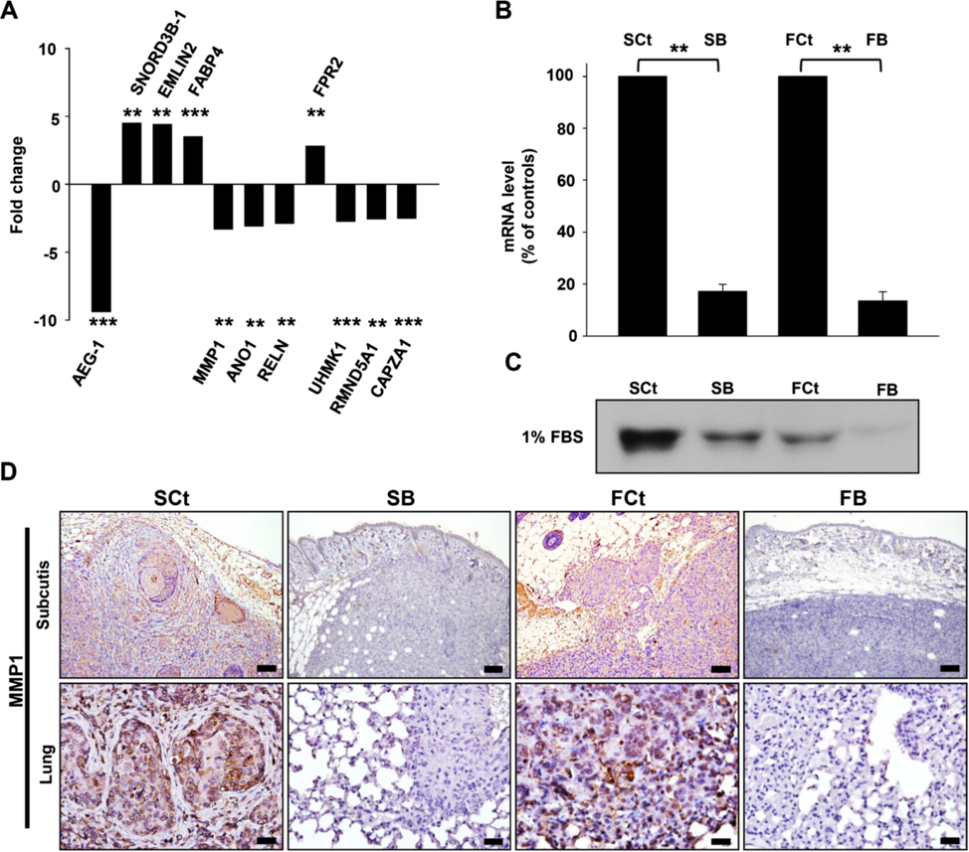
**AEG-1 knockdown down-regulated MMP1 expression in HNSCC cells *in vitro *and *in vivo*. (A)** microarray analysis of gene expression after AEG-1 knockdown in SAS cells. Genes with an absolute fold change greater than 2.5 are shown. SNORD3B-1, Homo sapiens small nucleolar RNA, C/D box 3B-1; EMILIN2, elastin microfibril interfacer 2; FABP4, fatty acid binding protein 4; MMP1, matrix metallopeptidase 1; ANO1, anoctamin 1; RELN, reelin; FPR2, formyl peptide receptor 2; UHMK1, U2AF homology motif kinase 1; RMND5A, required for meiotic nuclear division 5 homolog A; CAPZA1; capping protein (actin filament) muscle Z-line, alpha 1. *, *p* < 0.05; **, *p* < 0.01; ***, *p* < 0.001. **(B)** RT-QPCR confirmation of gene expression profiles after knockdown of AEG-1 in SAS and FaDu cells. All experiments were performed in triplicate (n = 3) and data were normalized to GAPDH. **(C)** secreted MMP1 protein in cell-conditioned culture media. Each cell lines were seeded in equal numbers (1 × 10^6^ cells) and were cultured in starving condition (1% fetal bovine serum, FBS) for 24 hr and the media were harvested. **(D)** immunohistochemical staining of MMP1 in murine subcutaneous xenograft tumors (upper row) and metastatic lesions from *in vivo* lung metastasis assays (lower row). Scale bar: upper row, 100 μm; lower row, 25 μm.

To determine the downstream targets of AEG-1 that contribute to invasion and metastasis pathways in HNSCC cells, we performed a microarray comparison between the gene expression profiles of SB cells and SCt cells. The expression level of MMP1 (matrix metalloproteinase 1) in SB cells was downregulated by approximately 3.3 fold, as compared to that in SCt cells (Figure 4A). RT-QPCR analysis also revealed a reduction in MMP1 mRNA in SB cells and FB cells (reduced to an average of 17.17% and 13.44% of the levels in the relevant controls, respectively, Figure 4B). In addition, AEG-1 knockdown caused a remarkable reduction of secreted MMP1 protein in the cell-conditioned culture media for both SAS and FaDu cells (Figure 4C). Immunohistochemical staining of MMP1 revealed a reduced positive signal in tumor xenografts and pulmonary metastatic lesions generated from AEG-1-knockdown HNSCC cells, as compared to the cytosolic and juxtacellular staining of MMP1 observed in lesions arising from control cells (Figure 4D). Also, incorporation of MMP inhibitor I (2 μM) hampered the invasion abilities of SAS and FaDu cells in transwell assays (Additional file 3: Figure S2). As MMPs are considered to be involved in both invasion and metastasis, MMP1 may be a downstream effector of AEG-1 in determining the aggressive phenotype of HNSCC.

### AEG-1 expression increases phosphorylation of the p65 subunit of NF-κB and enhances p65 binding to the MMP1 promoter

**Figure 5:**
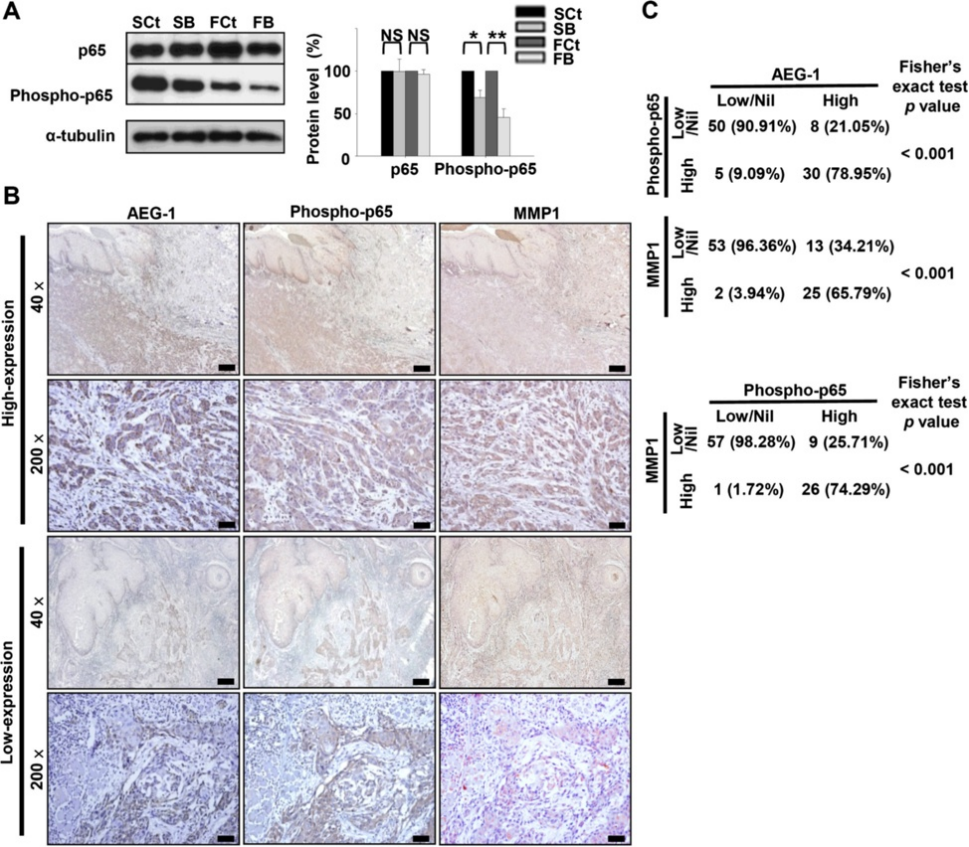
**AEG-1 knockdown suppressed phosphorylation of serine 536 of the p65 subunit (RelA) of NF-κB. (A)** Western blot of total p65 and phosphorylated p65 (serine 536). Data were normalized to respective controls and are were expressed as mean ± SEM; n = 3. NS, not significant (*p* > 0.05); *, *p* < 0.05; **, *p* < 0.01. **(B and C)** immunohistochemical staining of phosphorylated p65 (Ser536) and MMP1 in formalin-fixed, paraffin embedded OSCC specimens. Expression of phosphorylated p65 and MMP1 was positively cor related with that of AEG-1 both intratumorally **(B)** and intertumorally **(C)**. Scale bar: 40 ×, 300 μm; 200 ×, 45 μm.

We hypothesized that AEG-1 may affect MMP1 expression through NF-κB and AP1, since the promoter of MMP1 harbors regulation sites for these two transcription factors. Western blotting revealed that the levels of the phosphorylated p65 subunit of NF-κB (serine 536) in SB cells and FB cells were 68.84% and 45.64% that of the control counterparts (*p* = 0.013 and *p* = 0.005; *t*-test), respectively (Figure 5A). However, phosphorylation status of c-jun (a subunit of AP1), Akt and GSK3β (downstream targets of the PI3K pathway) were unaffected by AEG-1 knockdown (Additional file 4: Figure S3A). Also, the phosphorylation status of p65 at serine 468 and the level of phosphorylated IκB are unchanged after AEG-1 knockdown in HNSCC cell lines (Additional file 4: Figure S3B). These observations prompted us to examine the relationship between AEG-1 expression and the phosphorylation status of p65 at serine 536 in clinical specimens of HNSCC. A spatial correlation between AEG-1 and phosphorylated p65 was evident in the high AEG-1-expressing group, while the phosphorylated p65 signals in the low and nil AEG-1-expressing cases were primarily observed at the peripheral basal cells of the neoplastic nests (the location of AEG-1 proteins, Figure 5B). AEG-1, phosphorylated p65 and MMP1 were co-localized in the enrolled cohort of OSCC, and these associations were statistically significant (Figure 5C). Moreover, high levels of both phosphorylated p65 (serine 536) and MMP1 in neoplastic cells were positively associated with advanced tumor stages, as well as with regional lymph node metastasis in OSCC (Additional files 5 and 6: Tables S2 and S3). High MMP1 expression was also significantly associated with distant metastasis in our samples (Additional file 6: Table S3).

**Figure 6:**
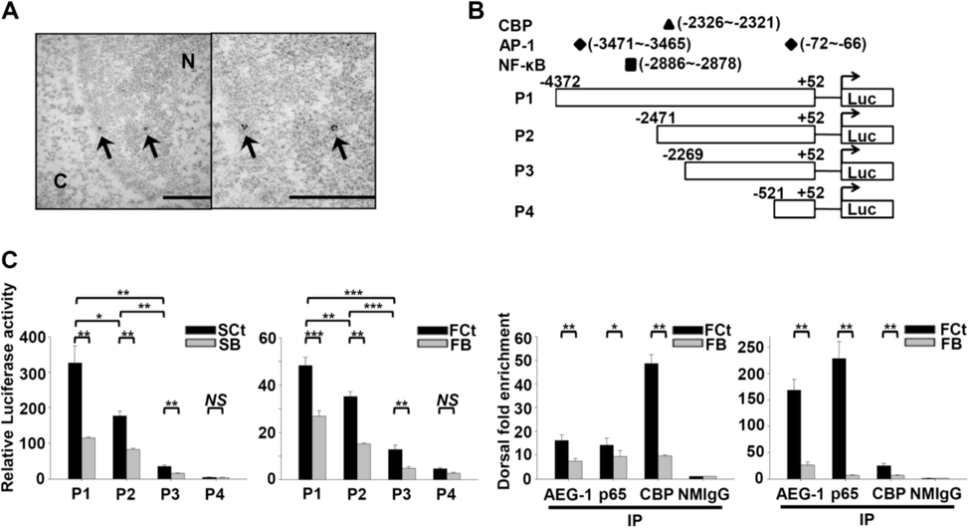
**AEG-1 regulates MMP1 expression at the transcriptional level through enhancing the binding of p65 to MMP1 promoter. (A)** nuclear translocation of AEG-1 protein was revealed by transmission electron microscopy. Arrow, 18 nm gold particles conjugated to anti-AEG-1 antibody. N, nucleus. C, cytosol. Right, magnified view. Scale bar, 500 nm. **(B)** constructs of the MMP1 promoter region used for the lucife-rase reporter assay. **(C)** transactivating activity of AEG-1 on MMP1 promoter constructs in the SAS and FaDu cell lines. All data were expressed as mean ± SEM; n = 3. **(D)** ChIP assay showing AEG-1, p65 and CBP binding to the NF-κB binding site of the MMP1 promoter in SAS and FaDu cell lines. NMIgG served as a negative control. All data were expressed as mean ± SEM; n = 3. NS, not significant (*p* > 0.05); *, *p* < 0.05; **, *p* < 0.01; ***, *p* < 0,001.

Transmission electron microscopy revealed nuclear translocation of AEG-1 protein (Figure 6A), suggesting that AEG-1 may not be restricted to the membrane and cytosol, as previously reported. To further validate the hypothesis that AEG-1 regulates MMP1 through NF-κB, we generated various luciferase reporter vectors driven by different fragments of the MMP1 promoter region (P1, -4372 to +52; P2, -2471 to +52; P3, -2269 to +52; and P4, -521 to +52) and transfected these reporters into the test cells (Figure 6B). A dramatic reduction of relative luciferase activity was observed in SB and FB cells transfected with P1 and P2, as compared to that in the respective controls; a smaller decrease was observed for knockdown cells transfected with P3, while luciferase activity was low in both knockdown and control cells transfected with P4 (Figure 6C). These results suggest that AEG-1 regulates elements between nucleotides -4372 to -2269 in the promoter of MMP1, where the binding sites of NF-κB and CBP are located. ChIP revealed that AEG-1, p65 and CBP binding were reduced at the NF-κB binding sequence of the MMP1 promoter in SB and FB cells as compared to that in the relevant controls, consistent with the data from our luciferase assay (Figure 6D). Taken together, these data suggest that AEG-1 increases phosphorylation of the p65 subunit of NF-κB and regulates the expression of MMP1 in HNSCC cells.

## Discussion

In this study, we found that high expression of AEG-1 was correlated with advanced tumor stages and regional lymph node metastasis in a large cohort of OSCC samples. The association between AEG-1 and distant metastasis was not statistically significant; evidence for an association may be confounded by the relatively low incidence (10%) of distant metastasis at initial presentation, a feature intrinsic to HNSCC [23]. In addition, our research has demonstrated that silencing of AEG-1 mitigates the malignant phenotypes of HNSCC cell lines *in vitro* and attenuates tumor growth and pulmonary metastasis *in vivo*. Our results provide the first strong evidence that AEG-1 is overexpressed in at least a subset of HNSCC and contributes to adverse clinical outcomes. Moreover, we found that AEG-1 upregulates the expression of MMP1, thereby uncovering a novel mechanism underlying the invasiveness of HNSCC.

Metastasis, defined as the detachment of daughter cells from the primary site of lesions and subsequent colonization of preferential target organs, is one of the hallmarks of malignancies [24,25]. The metastasis cascade can be divided into steps of local invasion, intravasation, survival, extravasation and colonization. Degradation and remodeling of extracellular matrix (ECM) are essential for neoplastic permeation into adjacent stomal tissue, as well as for breaching the perivascular basement membrane to initiate metastasis. MMPs are zinc-dependent enzymes, consisting of a propeptide, catalytic domain and a hemplexin-like C terminal domain. MMPs acquire enzymatic activity after peptidyl cleavage of the propeptide that interacts with the zinc ion in the catalytic domain [26]. The role of MMPs was traditionally believed to be primarily restricted to degradation of the ECM; however, mounting evidence suggests that MMPs are also involved in development, angiogenesis, inflammation and cancer progression, with the latter of which occurs through promoting migration and survival of cancer cells, orchestrating release of growth factors from extracellular reservoirs, and modulating recruitment of inflammatory cells to the tumor [27-29].

MMP1 is the stereotypical secreted collagenase of the MMP family, with its principle interstitial substrates consisting collagen I, II, III, VII, VIII, X, and gelatin. During collective cellular migration, which is the predominant pattern adopted by squamous cell carcinoma [30], MMP1 interacts with integrin α2β1 at the leading edge [31], and degrades native matrix macromolecules into fragments that are subsequently processed by the gelatinases MMP2 and MMP9 [32]. Analyses of clinical specimens revealed that expression of MMP1 correlated with lymphatic invasion and lymph node metastasis [33,34]. Furthermore, it is thought that MMP1 not only plays a pivotal role in vascular extravasation of metastatic neoplastic lesion, but it also contributes to the vascular remodeling at distant target sites, such as lung and bones [35-37]. Promotion of osteotropic metastasis can be accomplished in part by activation of the RANKL pathway through cleavage of EGF-like ligand by MMP1 [38]. In summary, MMP1 contributes to tumor invasion and metastasis by remodeling the matrix, and triggering the signaling cascades and crosstalk between neoplastic cells and adjacent interstitium. In our study, we have shown that AEG-1-knockdown SAS and FaDu cells reduce both the invasive ability of cancer cells (Figure 2D) and the expressions of MMP1 (Figure 4). Furthermore, MMP inhibitor is able to inhibit the invasive abilities of cancer cells (Additional file 3: Figure S2) to the level comparable to those observed in AEG-1-knockdown SAS and FaDu cells (Figure 2D). Taken together, these results indicate that AEG-1 is able to increase the invasive ability of cancer cells by increasing MMP1 expression.

To our knowledge, the present article is the first to report that AEG-1 regulates p65 phosphorylation at serine 536 and the subsequent MMP1 expression in HNSCC. MMP activity is modulated at various levels, including transcription, subcellular compartmentalization, proteolytic activation and inhibition. Data from our luciferase reporter assay indicate that AEG-1 regulates MMP1 transcription, primarily by acting on a region in the promoter upstream of nucleotide -2269. Previous studies reported the presence of NF-κB and AP-1 binding sites at nucleotides -2886 and -3471 of the MMP1 promoter, respectively [39,40]. Although AEG-1 has previously been reported to regulate MMP9 expression [41] through c-jun in human glioma cells [18], we found that AEG-1 does not affect phosphorylation of serines 63 and 73 of c-jun. On the other hand, AEG-1 knockdown in HNSCC cell lines attenuated phosphorylation of the p65 subunit (RelA) of NF-κB at serine 536. Phosphorylation at this amino acid residue is required for perinuclear localization of p65 to facilitate nuclear import [42,43]. This observation was supported by the finding that AEG-1 expression correlates with phosphorylation of p65 at serine 536, both intertumorally and intratumorally, in OSCC clinical specimens (*p* < 0.001, Fisher’s exact test). It was thought that the activation of NF-κB requires degradation of IκB. However, post-translational modifications of the subunits of NF-κB have also been found to determine the functional activity to a great extent. Serine 536 of p65, which is located within the C-terminal transactivation domain, is a target of multiple protein kinases, including IκB kinase α/β (IKKα/β), IKKϵ, TANK binding kinase 1 (TBK1), and ribosomal S6 kinase 1 (RSK1). Phosphorylation at this amino acid residue has also been reported to suppress nuclear export of NF-κB and to increase transactivation of a variety of downstream genes through positive interaction of p65 with co-activators (CBP and p300) [44-48]. Phosphorylation of serine 536 also activates the survival-promoting pathway when cells are challenged with chemotherapeutic cytotoxic agents such as doxorubicin and etoposide [43,49].

As mentioned previously, AEG-1 contains no known functional domains, making it unlikely that AEG-1 phosphorylates p65 directly. Based on our findings, it seems plausible that AEG-1 enhances p65 phosphorylation by recruiting associated protein kinases to the AEG-1-p65 complex. More detailed experiments are required to confirm this hypothesis. Our ChIP data also revealed that AEG-1 enhances the binding of p65 to the MMP1 promoter, thereby activating downstream genes by functioning as a linker between p65 and CBP to form the basal transcriptional machinery. Although p65 has been suggested to bind to AEG-1 through amino acid residues 101 to 205 of the latter [16], the interacting regions of CBP and AEG-1 remain unknown. Elucidation of the exact binding epitopes will require examination of the crystal structure of AEG-1 and its associated proteins.

## Conclusion

We used an extensive collection of HNSCC samples to demonstrate that elevated levels of AEG-1 protein are associated with lymph node metastasis and poor prognosis in HNSCC patients. AEG-1 primarily acts through the NF-κB pathway, by enhancing phosphorylation on serine 536 of RelA, which in turn results in an increased expression of MMP1. These findings may support the development of AEG-1-targeting therapeutics, such as small molecules, interference RNA with suitable drug delivering system or DNA vaccine, [50] to bolster our arsenal of adjuvant chemotherapy, and thereby improving the clinical outcome of HNSCC patients with high AEG-1 expression. In the meantime, elucidation of physiological function of AEG-1 through transgenic murine models and large-scale screening of the expression status of AEG-1 in normal human tissue are required to minimize the potential side effect.

## Methods

### Clinical specimen acquisition and clinicopathological staging

Formalin-fixed, paraffin-embedded OSCC tissue blocks were retrieved (79 men and 14 women, mean age 53.9 years, range 25–82 years) from the archive of the Department of Oral Pathology during 2000 to 2003, in accordance with ethical and institutional guidelines and with the approval of the Institutional Review Board of National Taiwan University Hospital (NTUH IRB, No 201111048RIC). Diagnosis of squamous cell carcinoma was based on histological examination of hematoxylin and eosin-stained (H&E) tissue sections by two qualified oral pathologists (YP Wang and CP Chiang). The pathological stage of each case at the time of surgery was determined according to the 7^th^ edition of staging criteria from the American Joint Committee on Cancer. Also, specimens of oral mucosa from healthy volunteer donors with informed consent (30 cases) were collected as controls during the extractions of their impacted wisdom teeth. Tissue sections, which included both tumor and adjacent non-tumor parts for comparison purposes, were cut to 4 μm in thickness.

### Immunohistochemical staining

Sections were deparaffinized and rehydrated, and antigen retrieval was performed concomitantly in the Trilogy buffer system (Cell Marque, Rocklin, CA, USA) in accordance with the manufacturer’s instructions. Endogenous peroxidase activity was then blocked by immersing the sections with 3% H_2_O_2_ in methanol for 30 minutes (min). After washing in phosphate-buffered saline (PBS), sections were incubated with 1% bovine serum albumin (BSA) for 30 min to block non-specific binding. Sections were then incubated with the monoclonal antibody Lyric 4–7 [51] at a concentration of 1 μg/ml for one hour at room temperature (RT). For staining of phosphorylated p65 and MMP1, anti-phosphorylated p65 polyclonal antibody (sc-101753, Santa Cruz biotechnology, TX, USA) and anti-human MMP1 antibody (clone 36665, R&D system, MN, USA) were used at a dilution of 1:50 and 15 μg/ml, respectively. After being washed in PBS containing 0.1% Tween 20 (PBST_0.1_), sections were treated with the polymer-based Super Sensitive IHC detection system (Biogenex, San Ramon, CA). In brief, sections were incubated with Super Enhancer reagent for 20 min at RT and were then thoroughly rinsed three times with PBST_0.1_ for 5 min each. Sections were subsequently treated with Poly-HRP reagent for 30 min at RT. Diaminobenzidine hydrochloride (DAB) (0.02%) containing 0.03% H_2_O_2_ was used as a chromogen to visualize peroxidase activity. The preparations were lightly counterstained with hematoxylin, mounted with Permount (Fisher Scientific, PA, USA), and examined by light microscopy. The population index (PI) was defined as: less than 10% positive tumor cells, 0; 10-49% positive tumor cells, 1; more than 50% positive tumor cells, 2. The intensity index (II) of the signal was designated as: none, 0; weak, 1; strong, 2. The labeling score (LS) was the product of PI and II for each case. Samples were futher categorized as: LS = 0, nil-expressing group; LS = 1 or 2, low-expressing group; and LS = 4, high-expressing group. Tissue sections incubated with normal mouse IgG (NMIgG) instead of primary antibody were used as negative controls. All histopathological images were taken with an Olympus BX51 microscope and DP2-BSW image acquisition software.

### Cell lines

OSCC cells lines HSC-3, SAS, and Ca9-22 were purchased from Japanese Collection of Research Bioresources Cell Bank and cell lines Cal 27 (OSCC) and FaDu (pharyngeal squamous cell carcinoma) were purchased from American Type Culture Collection. All cell lines were cultured in recommended media and were used in less than 6 months after resucscitation. The authenticity of SAS and FaDu cell lines was confirmed by STR profiling at Bioresource Collection and Research Center (BCRC) (Taiwan). Stable AEG-1 knock-down clones of SAS and FaDu cells (SA to SE cells and FA to FE cells, respectively) were established through transfection with lentiviral vectors carrying various AEG-1-specific shRNA sequences from the National RNAi Core Facility of Academia Sinica. As controls, stable clones transfected with scrambled shRNA were generated for each cell line (SCt cells and FCt cells, respectively). Stable clones were selected by treatment of the cells with 2 μg/ml puromycin for 14 days. Knockdown efficiency was determined by measuring mRNA and protein by real-time quantitative polymerase chain reaction (RT-QPCR) and Western blot, respectively (Additional file 2: Figure S1A and B).

### Western blot analysis

Western blots were performed using standard protocols, as previously described [51]. Total cell protein lysates of the indicated cell lines were loaded onto polyacrylamide gels supplemented with SDS (40 μg/lane). The following primary antibodies were used at the indicated concentrations: Lyric 4–7, 0.5 μg/ml; α-tubulin (Sigma-Aldrich Corporation, MO,USA), 5000-fold dilution; and MMP1 (clone 36665, R&D system, MN, USA), 1 μg/ml. Antibodies against the following proteins were purchased from Cell Signaling Technology (Danvers, MA, USA) and were used at a 1000-fold dilution: NF-κB (E498), phospho-NF-κB p65 (Ser536) (93H1), and phospho-NF-κB p65 (Ser468) (#3039).

### *In vivo* xenograft tumor assays

SCt, SB, FCt and FB cells were subcutaneously inoculated in pairs into the flanks of 6-week-old Nod/SCID mice (1 × 10^6^ cells/mouse, n = 6 per group). Laboratory animal husbandry and *in vivo* experiments were performed as per the guidelines of the National Laboratory Animal Center. The diameter of the resulting tumors were measured twice per week, and tumor volume was calculated as follows: large diameter × (small diameter)^2^  × 0.52. Xenograft tumors were harvested at the end point of the experiment and were sent for routine tissue processing.

### *In vivo* pulmonary metastasis assay

SCt, SB, FCt and FB cells were intravenously injected into six-week-old Nod/SCID mice (2 × 10^5^ cells/mouse, n = 10 per group) through the tail vein. All lung lobes were harvested twelve weeks later. Routine tissue processing was subsequently performed, and pulmonary metastatic foci were counted in sections stained with H&E.

### Microarray analysis

Total RNAs from SCt and SB cells were extracted and sent to the Microarray Core Facility of the Institute of Molecular Biology, Academia Sinica. The reverse- transcripted DNA probes were coupled with Alexa/CyDye and were hybridized with an Agilent human V2 GX array (44Kx4). The fluorescence of the array was scanned and analyzed, and the raw data were uploaded into the GEO data base as GSE 44766. Genes with a greater than 2.5 fold change (FC) in expression were then recorded (details in the Additional file 7: Supplementary Materials and Methods).

### Real-time quantitative polymerase chain reaction

Total RNA was extracted from SCt, SB, FCt and FB cells, and reverse transcription was subsequently performed. RT-QPCR was conducted using SYBR Green and the LightCycler480 II System (Roche Applied Science, Indianapolis, IN, USA). Each reaction was performed in triplicate (details in the Additional file 7: Supplementary Materials and Methods).

### Immunogold labeling and transmission electron microscopy

SAS cells were harvested and sent to the Electron Microscope Core Facility of the Institute of Cellular and Organismic Biology, Academia Sinica. Primary antibody against LYRIC (or NMIgG for negative controls) was applied at a concentration of 0.5 μg/ml for 1 hour at RT. The sections were examined with a transmission electron microscope (Hitachi H-7000) (details in the Additional file 7: Supplementary Materials and Methods).

### Luciferase assay of the MMP1 promoter region

The full length promoter region of the MMP1 gene [40] was cloned from of SAS cell genomic DNA using KAPA HiFi DNA polymerase with the following primers: CCCCTCGAGAGATGTAAGAGCTGGGAAAGGACGG (forward) and CCCAAGCTTTCAGTGCAAGGTAAGTGATGGCTTC (reverse). To generate a truncated fragment without the NF-κB binding site, the following primers were used: CCCCTCGAGCGATCTTCCATGAATACCTAACTGG (forward) and CCCAAGCTTTCAGTGCAAGGTAAGTGATGGCTTC (reverse). The cloned full length or truncated MMP1 promoters (nucleotides -2471 to +52) and luciferase reporter vector pGL4 (Promega, Madison, WI, USA) were digested with Xho I and HindIII. The full length or truncated promoter sequence was then ligated into pGL4 with T4 ligase at 16°C overnight. The resulting reporter vectors were designated as P1 (-4372 to +52) and P2 (-2471 to +52). Reporter vectors P3 (containing nucleotides -2269 to +52) and P4 (containing nucleotides -521 to +52) were generated by digesting the truncated MMP1 promoter either with SacI and HindIII or KpnI and HindIII, respectively, before ligating it into pGL4. Cells (SCt, SB, FCt and FB) were seeded onto 24-well plates (1 × 10^5^ cells/well) and were incubated at 37°C for 24 hours. The culture media was refreshed, and transfection with a reporter vector (one of P1 to P4) together with pGL4 (400 ng) and control *Renilla* vector (100 ng) was performed 30 min later, using Genejet (SignaGen laboratories, Rockville, MD) as per the manufacturer’s protocol. Firefly luciferase and Renilla readouts were acquired 18 hours post-transfection using the Dual-Glo Luciferase Assay System (Promega, Madison, WI, USA) according to the manufacturer’s recommendations.

### Chromatin immunoprecipitation (ChIP) assay

ChIP assay was performed with the Magna ChIP™ G kit (Millipore, Billerica, MA, USA) in accordance with the manufacturer’s protocol. In brief, 1 × 10^7^ cells were fixed with 1% paraformaldehyde and were lysed with cell lysis buffer and nuclear lysis buffer supplemented with protease cocktail inhibitors. Lysates were sonicated to shear the crosslinked DNA to a size of 200–1000 base pairs. Equal amounts of sheared cross-linked DNA were incubated at 4°C overnight on a shaker, with protein G magnetic beads coupled to either Lyric 4–7, anti-p65 antibody (L8F6, Cell Signaling, Danvers, MA, USA), anti-CREB binding protein (CBP) antibody (D9B6, Cell Signaling, Danvers, MA, USA) or NMIgG (as a negative control). The captured DNA-protein complexes were dissociated with elution buffer and proteinase K at 62°C for 2 hours with shaking. The free DNA fragments were then purified and quantified by RT-QPCR using primers (50 μM) flanking the p65 binding site (forward, GAGTTACAAAATTAAAACGGCTGA and reverse, CTGGCTGCTCTGTGAAAG). SYBR fluorescence was measured with a Lightcycler 480 II (Roche Applied Science, Indianapolis, IN, USA).

### Statistical analysis

The associations between clinicopathological parameters and the expression status of AEG-1, phosphorylated p65, and MMP1 within clinical specimens were analyzed by Fisher’s exact test. Disease-specific survival was compared between groups by Kaplan-Meier culmulative survival analysis with log-rank tests. All *in vitro* comparisons between HNSCC cells transfected with control shRNA and their experimental counterparts were performed with 3 technical replicates and 3 independent biological replicates (n = 3). Data analyses of *in vitro* experiments were performed by *t*-test. A *p*-value of less than 0.05 was considered statistically significant for all tests.

## Competing interest

The authors declare that they have no competing interest.

## Authors’ contributions

Conception and design: HCW and YPW. Development of methodology: YPW and IJL. Acquisition of data: YPW and IJL. Analysis and interpretation of data: YPW, IJL and CPC. Writing, review and/or revision of the manuscript: YPW and HCW. Administrative, technical, or material support: HCW. Study supervision: HCW. All authors read and approved the final manuscript.

## Supplementary Material

Additional file 1: Table S1 Correlation of advanced lymph node metastasis with AEG-1 in 93 cases of OSCC. Click here for file

Additional file 2: Figure S1 Establishment of stable clones of AEG-1-knockdown HNSCC cell lines. A, upper, sequences of shRNA targeting to AEG-1 mRNA; lower, AEG-1 protein expression in SAS cells after transfection of shRNA. B, Western blotting of total cell lysates from both cell lines transfected with lentiviral AEG-1-specific shRNA showed efficient AEG-1 suppression in protein level in SAS and FaDu cells (1.65% and 14.16% respectively, normalized with the expression levels of α-tubulin). Click here for file

Additional file 3: Figure S2 MMP inhibitor I (MMPInhI, 2 μM) impeded invasion of SAS and FaDu cells into Matrigel All values are the average of three independent experiments. ***, *p* < 0.001; **, *p* < 0.01. Scale Bar: 130 μm. Click here for file

Additional file 4: Figure S3 The phosphorylated status of components in PI3K pathway and NFκB pathway. A, c-jun and downstream effectors of PI3K pathway. B, IκB and p65 at amino acid residue serine 468. Click here for file

Additional file 5: Table S2 Clinicopathological correlation with phosphorylated p65 (serine 536) in 93 cases of OSCC. Click here for file

Additional file 6: Table S3 Clinicopathological correlation with MMP1 in 93 cases of OSCC. Click here for file

Additional file 7 Supplementary Materials and Methods. Click here for file
