# Supplementary material for: Astrocyte elevated gene-1 is associated with metastasis in head and neck squamous cell carcinoma through p65 phosphorylation and upregulation of MMP1
Source: Mol Cancer. 2013 Sep 24;12:109. doi: 10.1186/1476-4598-12-109 (PMC3856534; doi:10.1186/1476-4598-12-109)
Supplement: Additional file 1: Table S1 — Correlation of advanced lymph node metastasis with AEG-1 in 93 cases of OSCC. [file 1476-4598-12-109-S1.doc]

**Additional file 1:** Table S1

| **Correlation of advanced lymph node metastasis with AEG-1**  **in 93 cases of OSCC** | | | |
| --- | --- | --- | --- |
| **Parameter** | **AEG-1 expression status** | | **Fisher’s exact test**  ***p* value** |
|  | **Low**  **No. (%)** | **High**  **No. (%)** |  |
| **N** |  |  |  |
| N0+N1 | 50 (90.91%) | 26 (68.42%) | 0.012 |
| N2+N3 | 5 (9.09%) | 12 (31.58%) |  |
