# Supplementary material for: Astrocyte elevated gene-1 is associated with metastasis in head and neck squamous cell carcinoma through p65 phosphorylation and upregulation of MMP1
Source: Mol Cancer. 2013 Sep 24;12:109. doi: 10.1186/1476-4598-12-109 (PMC3856534; doi:10.1186/1476-4598-12-109)
Supplement: Additional file 2: Figure S1 — Establishment of stable clones of AEG-1-knockdown HNSCC cell lines. A, upper, sequences of shRNA targeting to AEG-1 mRNA; lower, AEG-1 protein expression in SAS cells after transfection of shRNA. B, Western blotting of total cell lysates from both cell lines transfected with lentiviral AEG-1-specific shRNA showed efficient AEG-1 suppression in protein level in SAS and FaDu cells (1.65% and 14.16% respectively, normalized with the expression levels of α-tubulin). [file 1476-4598-12-109-S2.doc]

**Additional file 2: Figure S1**. Establishment of stable clones of AEG-1-knockdown HNSCC cell lines. A, upper, sequences of shRNA targeting to AEG-1 mRNA; lower, AEG-1 protein expression in SAS cells after transfection of shRNA. B, Western blotting of total cell lysates from both cell lines transfected with lentiviral AEG-1-specific shRNA showed efficient AEG-1 suppression in protein level in SAS and FaDu cells (1.65% and 14.16% respectively, normalized with the expression levels of α-tubulin).
