# Supplementary material for: Astrocyte elevated gene-1 is associated with metastasis in head and neck squamous cell carcinoma through p65 phosphorylation and upregulation of MMP1
Source: Mol Cancer. 2013 Sep 24;12:109. doi: 10.1186/1476-4598-12-109 (PMC3856534; doi:10.1186/1476-4598-12-109)
Supplement: Additional file 3: Figure S2 — MMP inhibitor I (MMPInhI, 2 μM) impeded invasion of SAS and FaDu cells into Matrigel All values are the average of three independent experiments. ***, p < 0.001; **, p < 0.01. Scale Bar: 130 μm. [file 1476-4598-12-109-S3.doc]

**Additional file 3: Figure S2.** MMP inhibitor I (MMPInhI, 2 μM) impede invasion of SAS and FaDu cells into Matrigel All values are the average of three independent experiments. ***, *p* < 0.001; **, *p* < 0.01. Scale Bar: 130 μm.
