# Supplementary material for: Astrocyte elevated gene-1 is associated with metastasis in head and neck squamous cell carcinoma through p65 phosphorylation and upregulation of MMP1
Source: Mol Cancer. 2013 Sep 24;12:109. doi: 10.1186/1476-4598-12-109 (PMC3856534; doi:10.1186/1476-4598-12-109)
Supplement: Additional file 4: Figure S3 — The phosphorylated status of components in PI3K pathway and NFκB pathway. A, c-jun and downstream effectors of PI3K pathway. B, IκB and p65 at amino acid residue serine 468. [file 1476-4598-12-109-S4.doc]

**Additional file 4: Figure S3**. The phosphorylated status of components in PI3K pathway and NFκB pathway. A, c-jun and downstream effectors of PI3K pathway. B, IκB and p65 at amino acid residue serine 468.
