# Supplementary material for: Astrocyte elevated gene-1 is associated with metastasis in head and neck squamous cell carcinoma through p65 phosphorylation and upregulation of MMP1
Source: Mol Cancer. 2013 Sep 24;12:109. doi: 10.1186/1476-4598-12-109 (PMC3856534; doi:10.1186/1476-4598-12-109)
Supplement: Additional file 5: Table S2 — Clinicopathological correlation with phosphorylated p65 (serine 536) in 93 cases of OSCC. [file 1476-4598-12-109-S5.doc]

**Additional file 5:** Table S2

| **Clinicopathological correlation with phosphorylated p65**  **(serine 536) in 93 cases of OSCC** | | | |
| --- | --- | --- | --- |
| **Parameter** | **pP65 expression status** | | **Fisher’s exact test**  ***p* value** |
|  | **Low**  **No. (%)** | **High**  **No. (%)** |  |
| **Stage** |  |  |  |
| I+II | 32 (55.17%) | 8 (22.86%) | 0.003 |
| III+IV | 26 (44.83%) | 27 (77.14%) |  |
| **T** |  |  |  |
| T1+T2 | 34 (58.62%) | 19 (54.29%) | 0.829 |
| T3+T4 | 24 (41.38%) | 16 (45.71%) |  |
| **N** |  |  |  |
| N0 | 50 (86.21%) | 14 (40.00%) | <0.001 |
| N1+N2+N3 | 8 (13.79%) | 21 (60.00%) |  |
| **M** |  |  |  |
| M0 | 57 (98.28%) | 31 (88.57%) | 0.065 |
| M1 | 1 (1.72%) | 4 (11.43%) |  |
| **Recurrence** |  |  |  |
| Negative | 47 (81.03%) | 28 (80.00%) | 1.000 |
| Positive | 11 (18.97%) | 7 (20.00%) |  |
| **Differentiation** |  |  |  |
| Well | 51 (87.93%) | 24 (68.57%) | 0.03 |
| Moderate/poor | 7 (12.07%) | 11 (31.43%) |  |
