# Supplementary material for: Astrocyte elevated gene-1 is associated with metastasis in head and neck squamous cell carcinoma through p65 phosphorylation and upregulation of MMP1
Source: Mol Cancer. 2013 Sep 24;12:109. doi: 10.1186/1476-4598-12-109 (PMC3856534; doi:10.1186/1476-4598-12-109)
Supplement: Additional file 6: Table S3 — Clinicopathological correlation with MMP1 in 93 cases of OSCC. [file 1476-4598-12-109-S6.doc]

**Additional file 6:** Table S3

| **Clinicopathological correlation with MMP1 in 93 cases of OSCC** | | | |
| --- | --- | --- | --- |
| **Parameter** | **MMP1 expression status** | | **Fisher’s exact test**  ***p* value** |
|  | **Low**  **No. (%)** | **High**  **No. (%)** |  |
| **Stage** |  |  |  |
| I+II | 36 (54.55%) | 4 (14.81%) | <0.001 |
| III+IV | 30 (45.45%) | 23 (85.19%) |  |
| **T** |  |  |  |
| T1+T2 | 40 (60.61%) | 13 (48.15%) | 0.357 |
| T3+T4 | 26 (39.39%) | 14 (51.85%) |  |
| **N** |  |  |  |
| N0 | 55 (83.33%) | 9 (33.33%) | <0.001 |
| N1+N2+N3 | 11 (16.67%) | 18 (66.67%) |  |
| **M** |  |  |  |
| M0 | 65 (98.48%) | 23 (85.19%) | 0.024 |
| M1 | 1 (1.52%) | 4 (14.81%) |  |
| **Recurrence** |  |  |  |
| Negative | 60 (90.91%) | 22 (81.48%) | 0.287 |
| Positive | 6 (9.09%) | 5 (18.52%) |  |
| **Differentiation** |  |  |  |
| Well | 57 (86.36%) | 18 (66.67%) | 0.042 |
| Moderate/poor | 9 (13.94%) | 9 (33.33%) |  |
