# Supplementary material for: Astrocyte elevated gene-1 is associated with metastasis in head and neck squamous cell carcinoma through p65 phosphorylation and upregulation of MMP1
Source: Mol Cancer. 2013 Sep 24;12:109. doi: 10.1186/1476-4598-12-109 (PMC3856534; doi:10.1186/1476-4598-12-109)
Supplement: Additional file 7 — Supplementary Materials and Methods. [file 1476-4598-12-109-S7.doc]

**Supplementary Materials and Methods**

***Antibodies*** ***for Western blots***

Antibodies against the following proteins were purchased from Cell Signaling Technology (Danvers, MA, USA) and used at a 1000-fold dilution: c-Jun (60A8), phospho-c-Jun (Ser63) (54B3), phospho-c-Jun (Ser73) (#9164), pan-Akt (C67E7), phospho-Akt (Ser473) (D9E), phospho-Akt (Thr308) (C31E5), phospho-GSK-3β (5B3), phospho-c-Raf, IκBα (L35A5), phospho-IκBα (serine32) (14D4), and phospho-NF-κB p65 (Ser468) (#3039)

**WST-1 assays**

Cells were seeded in 96 well-plates at a concentration of 3 × 10^3^ cells/well with matched media. One day later, 4-[3-(4-iodophenyl)-2-(4-nitrophenyl)-2H-5-tetrazolio]-1,3-benzene disulfonate (WST-1, Roche) was added to every well at a concentration of 0.5 mg/ml, and the cells were incubated for 3 hours at 37°C. After incubation, the absorbance of the reaction products at a wavelength of 440 nm was measured, and considered as the absorbance for Day 0. The procedure was repeated each day for the next four days (Day 1 to Day 4).

**Colony formation assays**

Cells were inoculated in 6 cm dishes (1 × 10^3^ cells/dish) and kept at 37°C for 10 days. The colonies that formed were fixed with 4% paraformaldehyde for 5 min, and then stained with 1% crystal violet for 20 min. Colonies larger than 1 mm in diameter were counted and the total number recorded.

**Migration assays and transwell Matrigel invasion assays**

For the migration assay, SCt, SB, FCt and FB cells were added to each well with culture-inserts (Ibidi, Martinsried, Germany) (1 × 10^4^ cells/well) and incubated at 37°C with 5% CO_2_. Confluence was achieved 36 hours later. The inserts were then removed, and the initial gaps were 500 μm wide. Serial photographs were taken at 4 hour intervals. For the invasion assay, Matrigel basement membrane matrix (BD Biosciences, San Jose, CA, USA) was coated to the upper side of hanging cell culture inserts (Millipore, Billerica, MA, USA) at a concentration of 2 mg/ml. Cells were seeded into the coated hanging inserts (2 × 10^5^ cells/insert) and incubated with the corresponding culture media. The lower chamber of the invasion system was filled with serum-free culture media. The cells and Matrigel on the upper side of the inserts were removed after 24 hours. The migrated cells on the lower surface of the inserts were fixed with methanol and counted after visualization with 10 fold diluted Giemsa stain. In MMP inhibitor transwell assays, MMP inhibitor I (MMPInhI, Merck) was incorporated into corresponding culture media at a concentration of 2 μM in the hanging inserts and the diluted Matrigel.

**Microarray analysis**

Total RNA from 1 × 10^7^ SCt or SB cells was extracted with 1 ml TRI reagent (Invitrogen, Carlsbad, CA, USA) and incubated for 5 min at RT. Two hundred microliters of chloroform were added to the RNA, and the mixture was shaken vigorously for 15 seconds before being incubated for 5-15 min at RT. The mixtures were then subjected to centrifugation at 12,000 *g* for 10-15 min at 4°C. The aqueous phase was transferred to a fresh tube and incubated with 500 μl isopropanol for 5-10 min at RT. After centrifugation at 12,000 *g* for 10 min at 4°C, the supernatants were discarded and the RNA pellets washed with 1 ml 75% ethanol. The ethanol was then removed and the RNA pellets dissolved in 20 μl H_2_O. RNA extracts from SCt and SB cells were sent to the Microarray Core Facility of the Institute of Molecular Biology, Academia Sinica. Reverse transcription with aa-dUTP labeling was performed, and the associated RNA was subsequently degraded and removed. The resulting DNA probes were coupled with Alexa/CyDye, washed and hybridized with an Agilent human V2 GX array (44Kx4).

**Real-time quantitative polymerase chain reaction**

Total RNA was extracted from SCt, SB, FCt and FB cells as described above. Reverse transcription was performed using 4 μg aliquots of total RNA with Super-Script III RNase H-reverse transcriptase (Invitrogen, Carlsbad, CA, USA) and oligo (dT) primers (Fermentas, Glen Burnie, MD, USA), as per the manufacturer’s instructions. RT-QPCR was performed using SYBR Green and the LightCycler480 II System (Roche Applied Science, Indianapolis, IN, USA). The primers used were as follows: AEG-1, GAAGAAGCAAGGTGAAGATAACT (forward) and TTGGACGGGTTTTAGAGGTATT (reverse); MMP1, CTGGAAGGGCAAGGACTCTA (forward) and CTTCCCAGCCTCTTGCTG (reverse). The expression levels of each gene were normalized to that of GAPDH in the same sample. Each reaction was performed in triplicate.

**Immunogold labeling and transmission electron microscopy**

SAS cells were harvested (1 × 10^7^ cells) and sent to the Electron Microscope Core Facility of the Institute of Cellular and Organismic Biology, Academia Sinica, for sample preparation and ultrathin sectioning. The sections were then washed with Tris-buffered saline (TBS) for 15 min, and blocked with 1% BSA for 15 min. Primary antibodies against AEG-1 (or NMIgG for negative controls) was applied at a concentration of 0.5 μg/ml for 1 hour at RT. Sections were then washed four times with TBS (5 min/wash), before being incubated with secondary anti-mouse IgG conjugated to 18 nm gold particles for 1 hour at RT. After incubation, the sections were thoroughly washed four times with TBS (5 min/wash) and five times with ddH_2_O (5 min/wash). Next, the sections were treated with 5% uranyl acetate in H_2_O for 10 min, and then with 0.5% lead citrate for 4 min before being completely air dried. The sections were examined with a transmission electron microscope (Hitachi H-7000).
